# Erector Spinae Plane Block for the Treatment of Post Mastectomy Pain Syndrome

**DOI:** 10.7759/cureus.12656

**Published:** 2021-01-12

**Authors:** Jamal Hasoon, Ivan Urits, Omar Viswanath, Bilal Dar, Alan D Kaye

**Affiliations:** 1 Anesthesiology • Pain Management, Beth Israel Deaconess Medical Center and Harvard Medical School, Boston, USA; 2 Anesthesiology • Pain Medicine, Beth Israel Deaconess Medical Center and Harvard Medical School, Boston, USA; 3 Anesthesiology • Pain Medicine, Louisiana State University Health Sciences Center, Shreveport, USA; 4 Anesthesiology • Chronic Pain Management, Brigham and Women's Hospital and Harvard Medical School, Boston, USA; 5 Pain Management, Louisiana State University Health Sciences Center, Shreveport, USA

**Keywords:** post mastectomy pain syndrome, breast surgery, chronic pain, erector spinae plane block

## Abstract

Postmastectomy pain syndrome (PMPS) is a common complication after breast cancer surgery and is often challenging to manage. Treatment options include medication management, physical therapy, and interventional procedures. The erector spinae plane block (ESPB) is a regional technique proven to help both acute postoperative analgesia and chronic neuropathic pain conditions. This block is becoming more popular in the chronic pain setting for neuropathic thoracic pain conditions. We describe the utilization of the ESP block for significant neuropathic breast pain after total mastectomy. Our case demonstrates the utility of this block for women suffering from severe PMPS.

## Introduction

Postmastectomy pain syndrome (PMPS) is a common complication after breast cancer surgery and is often challenging to manage. It is estimated that PMPS occurs in 20%-44% of patients after breast surgery [1,2]. Treatment options for this painful condition include medication management, physical therapy, and interventional management. Medications typically include gabapentinoids, antidepressants, nonsteroidal anti-inflammatory drugs (NSAIDs) and opioid pain medications. Physical therapy often is included to help improve overall physical function and mobility. When conservative therapy proves ineffective, interventional pain procedures should be employed as a modality to help with PMPS.

Various interventional procedures have been utilized, including intercostal nerve blocks, paravertebral blocks, and serratus plane blocks with varying success [3,4]. Alternatively, the erector spinae plane block (ESPB) is a regional technique that can be utilized to provide analgesia for various thoracic neuropathic pain conditions. We describe the case of a woman with severe PMPS who obtained significant relief with a single ESPB.

## Case presentation

The patient was a 63-year-old female who had undergone a total mastectomy six years before breast cancer treatment. She reported severe allodynia and constant burning pain in her left breast. She noted the pain was debilitating, and she was unable to wear any tight-fitting clothing or use a blanket over her chest at night. She reported a pain level of 9/10 intensity on a numerical rating scale (NRS). The patient also endorsed that the pain frequently disrupted her sleep. She had tried several medications, including acetaminophen, NSAIDs, lidocaine patches, gabapentin, pregabalin, and was currently being managed with opioid pain medications with mild improvement. The patient consented for an ESPB to assist with her chronic pain condition.

**Figure 1:**
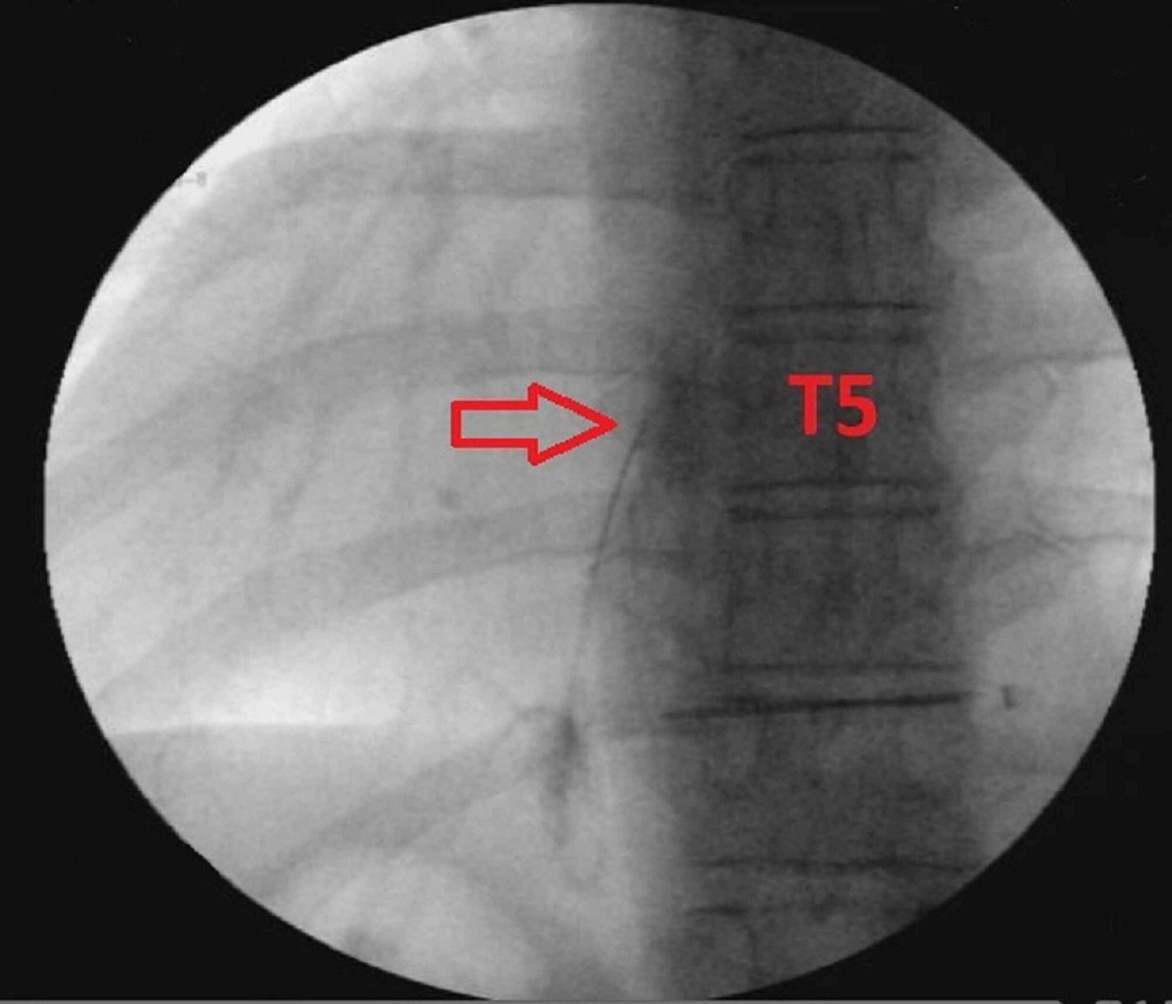
Erector spinae plane block with fluoroscopic guidance Fluoroscopy was used to confirm the correct level for the block. The red arrow is pointing towards the transverse process, which is the target for this block.

The patient underwent the procedure under fluoroscopic guidance. A 25-gauge spinal needle was advanced until it contacted the transverse process at the T5 level. Next, 2mL of nonionic contrast was injected to confirm that the injected medications would not be deposited intravascularly. Then a solution of 1mL of methylprednisolone 40mg/mL with 9mL of 0.25% bupivacaine was injected to perform the ESPB (Figure 1). The procedure was performed without complications.

## Discussion

The patient reported significant relief shortly after the procedure. She noted that her pain in the recovery area after the procedure was 0/10 on NRS. At her one month follow up appointment she reported she still had a 70% improvement of her pain and had decreased her opioid usage by 50%. She continues to have pain relief three months following the procedure and reports significant improvement in her quality of life. Additionally, she has continued to do well with her decrease in opioid requirements and has not needed to escalate her pain medications dose.

The ESPB can be utilized to provide analgesia for both acute and chronic pain conditions. The block allows for the spread of medication along the fascial plane, which anaesthetizes somatic nerves. The ESPB has traditionally been utilized for acute postoperative pain control [5,6]. The use of the ESPB for chronic pain has recently expanded for various neuropathic pain conditions [7-9]. The ESPB is relatively new as it was first reported in 2016 to manage thoracic neuropathic pain in a patient with metastatic disease with rib fractures [10].

This block usage has expanded dramatically for postoperative pain and is now gaining popularity for chronic neuropathic pain conditions. The procedure can be performed with either fluoroscopy or ultrasound guidance and is a relatively safe procedure with a wide variety of applications. [8,11] Given that PMPS is such a common and debilitating complication after breast surgery, the ESPB should be considered for patients who do not respond to conservative therapy. Additionally, this procedure should be considered for any patient suffering from neuropathic thoracic pain conditions.

## Conclusions

PMPS is a common and debilitating complication from breast surgery. The ESPB can be utilized to provide analgesia for patients suffering from this difficult to manage the condition. Our case demonstrates the ease and utility of this procedure and the promising results reported from our patient. Given that PMPS is a relatively common complication, physicians treating these patients should be aware of patients' options suffering from this chronic pain condition.
